# Abstract of the Proceedings of the Buffalo Medical Association

**Published:** 1861-09

**Authors:** J. B. Samo

**Affiliations:** Secretary pro tem.


ART. II.—*Abstract of the Proceedings of the Buffalo Medical Association.*

TUESDAY EVENING, August 6th, 1861.

The Association met at the usual hour.

Present—Dr. C. C. F. Gay, President, in the Chair; Drs. Rochester, Ring, White, Eastman, Congar, Treat, Cronyn, Samo.

The reading of the minutes of the last meeting were dispensed with.

Propositions for membership being in order, Drs. C. P. Fanner and George Sweet were proposed for election as members.

*Dr. White* related the following case of spontaneous luxation of the hip joint:

*History*—Mrs. C——, aged 28 years, one of a family of five children, all apparently healthy; parents healthy and free from from any known tendency to disease. Mrs. C—— had ague from her tenth to her thirteenth year very severely; menstruated at sixteen; menstruation unfrequent and scanty, with from three to six months' intermission. Married at nineteen years of age. Pregnant eigeteen months after marriage. Aborted at second month. Health very infirm after this period, with constant leucorhœal discharge; during last two years suffered greatly with rheumatism or neuralgia; it had been differently regarded by her former medical attendants, but at no time was there swelling of the joints, or any appearance of articular rheumatism. In January, 1860, became much more feeble, and right hip and leg more painful; pain increased considerably by exercise' and she soon became bed-ridden; the thigh was flexed upon the pelvis, and while in this position she suffered from a spasmodic action of the muscles which produced most excruciating pain. She was greatly emaciated; had never received any fall or injury. In October, 1860, was brought to this city and placed under my care. Upon examination the head of os femoris was found to rest upon dorsum of illium; the dislocation was complete. By measurements made by Prof. E. M. Moore of Rochester, and myself, from all points, both on the anterior and posterior surfaces of the pelvis, this diagnosis was fully confirmed. Head of os femoris two to two and one-half inches out of natural position; extremity shortened; heel elevated; toe turned towards opposite instep; all signs of this form of dislocation unmistakably present; general health very feeble; had to be brought to the city upon a bed; uterine symptoms prominent; examination with speculum shows os uteri ulcerated; neck elongated and greatly enlarged. Local treatment, caustics and iodine to the os uteri. Internally, iron, quinine, stimulants and nutritious diet.

The head of the bone having been probably more than six months out of its place, no effort at reduction was made; this condition of the hip joint had not until now been suspected. The patient was encouraged to use the joint, making motion and walking with it as much as possible. She was therefore given some crutches and directed to use the limb; commenced to improve in appetite and strength; has gained thirty pounds in flesh; finally, general health is fully restored; walks by aid of her crutches half a mile; has the gait and general appearance of a dislocated femur. Menstruation returned in January last, and has been regular ever since, though never so before during her life. Hypertrophy of uterus reduced, ulcerations healed, leucorrhœa wholly disappeared. She is rapidly improving in her ability to use the leg, take exercise, &c.

*Remarks.*—This case of spontaneous luxation of the hip presents some points of especial interest, since it occurred without any evidences of scrofula or other disease. 1st—How shall we account for this displacement? Perhaps we cannot fully and satisfactorily explain it. Muscular contraction or irregular muscular action is the only conceivable force which could have acted to produce this result, and yet it does not readily and plainly appear how any of the muscles of the hip or thigh could possibly act to draw the head of the thigh bone out of its natural bed. Persons not unfrequently possess the power of dislocating *partially* certain joints by voluntary muscular action, but however strongly they exert their will they cannot produce anything like complete luxation of the hip joint. Dr. Haynes of Saratoga, N. Y., has recently published the particulars of the case of a lad, aged seven years, who is able to dislocate, and also to reduce the joints of the knee, elbow, wrist, thumb and fingers, with perfect ease by muscular contraction. Of the ability of the muscles to produce such results, experience has furnished ample proof; the shoulder, lower jaw, and other orbicular joints are often displaced by muscular action. Several cases have been recorded of displacement of the thigh bone, but it must be a very rare occurrence for complete dislocation of the hip to be produced by muscular contraction alone. The great relaxation of the system induced by long continued pain and disease, together with the flexed condition of the thigh, and the violent spasmodic action of the muscles, constitutes the best explanation which I can suggest of the displacement found to exist in this most remarkable case.

A point of great interest and worthy most careful consideration, is the apparent induction of spasmodic action in the muscular system by various uterine diseases. It will be asked if the disease found to exist in the neck and os uteri did not really constitute all the primary affection producing the so called rheumatism, neuralgia, &c., inducing the leucorrhœa and spasmodic (hysterical) muscular action, and through it the dislocation, debility, and in short the whole train of diseased action.

*Dr. Samo* described a case of vaginitis and conjunctivitis in a girl five years of age. When the child came under his care, which was over a fortnight after disease had commenced, there was a pro fuse discharge from the vagina, with deep redness of its mucous lining, with ardor, urinæ, &c. The right eye was closed, the lids red, and so tumefied from serous effusions, that the child could not open them. Upon separating them, after wiping away the yellow discharge, the conjunctiva was found swollen into folds, so as nearly to conceal the c ornea, exhibiting the condition named chemosis, in a remarkable degree; this latter membrane being clear, at least what was visible of it. Some intolerance of light existed, though not extreme. There was considerable febrile excitement, hot and dry skin, pain in the head, loss of appetite, &c. The child was of a fair complexion, and evidently of a scrofulous diathesis. There was no evidence of any venereal cause that could be discovered, although the occurrence of the ophthalmia might, perhaps, create a suspicion. The treatment consisted of the warm bath daily, the steady application of cold water to the eye for four or five days, followed by the nit. silver ij. grains to the one oz. water, and the internal administration of calomel, one-fourth grain combined with ipecac and quinine every six hours. Under this treatment the patient steadily improved, and at this time, less than a fortnight since commencing it, the child has nearly recovered its usual health.

Some discussion ensued as to the cause in cases of this kind in children, and evidence given to prove their venereal origin in many instances.

*Dr. Ring* spoke of a case of gonorrhœa in a child ten years old.

*Dr. Rochester* reported a case of premature development amounting to monstrosity. A German girl three years of age was brought to him by her parents. She was as large as most children at four years. The mammæ were of the size seen in healthy girls of sixteen or seventeen, very prominent and conical. The external sexual organs were also large, and the mons veneris had a heavy downy covering. The child could not yet talk. She had brothers and sisters, none of whom presented any unusual sexual or mammaary enlargement; she did not menstruate.

*Dr. Treat* stated that there was a child about five years of age, near his residence, who had a singular arrest of of development. His legs were stopped in growth at the knee, save that one of them had something more like a finger than a toe growing therefrom.

*Dr. Congar* enquired whether eruption followed diphtheria; recently a case of his had eruption of a peculiar character.

*Dr. Rochester* answered, that it was a question which had been asked by others, as by Dr. Alonzo Clark, &c.

*Reports of prevailing diseases.*—*Dr. Ring* mentioned a case of diphtheria as seen to-day. Dr. Cronyn mentioned some twenty cases diarrhœa during the past week. Dr. Rochester mentioned whooping cough as prevalent. Dr. Eastman noticed a tendency to summer complaints.

*Dr. Treat* presumed, as he had just returned from Washington, and assisted in dressing the wounded which were brought into, or came into, Fort Runyon, on Monday, the 22d July, after the battle of Bull's Run, (Manassas,) this Society would be interested to hear a report in brief. They need not expect to hear of mangled corpses, shattered bones and many amputations, (capital operations,) which modern device had brought into the diablery of human destruction. The fact was, that although at Fort Runyon probably one-fifth of the wounded were dressed, that escaped the enemy, not a single amputation was performed of any magnitude. One amputation, near the shoulder, well dressed, arrived from the field, two shattered fore-arms put in temporary splints were dressed, three or four slight shell wounds or bruises, one or two slight sabre and bayonet wounds, the rest were chiefly bullet wounds, passing through the calf, or the thick of the thigh, arm, shoulder, near the axillor, and across the flexed elbow. These latter were the most numerous, and on investigating the position in drill, I believe they were received in the "aim," preparatory to "fire." Honorable wounds these latter, and I think all of them. These wounded, all, or nearly all, came to the Fort on foot; walking or running fifteen to thirty miles, with few exceptions where a horseman took a lame rider on behind.

The whole number dressed were from one hundred to one hundred and twenty-five. By order of Dr. Wilcox, the faithful Surgeon of the Twenty-First Regiment, a record was kept of the names, residences, Regiment, &c. The Assistant, Dr. Peters, and Dr. Marsh of the Third New Jersey, were actively employed, and Dr. Frank H. Hamilton an half hour in the forenoon. From the ambulances which passed over supposing I should see what a surgeon would like to see, (if to be seen,) *capital operations.* I left Fort Runyon at night, (the wounded being all dressed,) in a violent rain storm, and on Tuesday obtained access to the City Hospital. I was surprised to find that the cases were very similar to those in Fort Runyon, and to learn that in other Hospitals the cases were also parallel.

J. B. Samo, *Secretary pro tem.*

